# Current management of patients hospitalized with community-acquired pneumonia across Europe: outcomes from REACH

**DOI:** 10.1186/1465-9921-14-44

**Published:** 2013-04-15

**Authors:** Francesco Blasi, Javier Garau, Jesús Medina, Marco Ávila, Kyle McBride, Helmut Ostermann

**Affiliations:** 1 Department of Pathophysiology and Transplantation, Università degli Studi di Milano, IRCCS Fondazione Ca’ Granda, Ospedale Maggiore Policlinico, Via Francesco Sforza 35, 20122, Milan, Italy; 2 Department of Medicine, Hospital Universitari Mutua de Terrassa, Plaza Doctor Robert 5, Terrassa, Barcelona, 08221, Spain; 3 Medical Evidence Centre (Global Medical Affairs), AstraZeneca, Parque Norte, Edificio Roble, Serrano Galvache 56, Madrid, 28033, Spain; 4 AstraZeneca EMEA, Level 7, 2 Kingdom Street, London, W2 6BD, UK; 5 Instat Services, Inc., 1 Wilson Street, Chatham, NJ, 07928, USA; 6 Department of Internal Medicine III, Haematology and Oncology, University Hospital Munich, Munich, Germany

**Keywords:** Antibacterial agents, Clinical management, Community-acquired pneumonia, Retrospective studies

## Abstract

**Background:**

Data describing real-life management and treatment of community-acquired pneumonia (CAP) in Europe are limited. REACH (http://NCT01293435) was a retrospective, observational study collecting data on the management of EU patients hospitalized with CAP.

The purpose of this study was to understand patient and disease characteristics in patients hospitalized with CAP and to review current clinical practices and outcomes.

**Methods:**

Patients were aged ≥18 years, hospitalized with CAP between March 2010 and February 2011, and requiring in-hospital treatment with intravenous antibiotics. An electronic Case Report Form was used to collect patient, disease and treatment variables, including type of CAP, medical history, treatment setting, antibiotics administered and clinical outcomes.

**Results:**

Patients (N = 2,039) were recruited from 128 centres in ten EU countries (Belgium, France, Germany, Greece, Italy, the Netherlands, Portugal, Spain, Turkey, UK). The majority of patients were aged ≥65 years (56.4%) and had CAP only (78.8%). Initial antibiotic treatment modification occurred in 28.9% of patients and was more likely in certain groups (patients with comorbidities; more severely ill patients; patients with healthcare-associated pneumonia, immunosuppression or recurrent episodes of CAP). Streamlining (de-escalation) of therapy occurred in 5.1% of patients. Mean length of hospital stay was 12.6 days and overall mortality was 7.2%.

**Conclusion:**

These data provide a current overview of clinical practice in patients with CAP in EU hospitals, revealing high rates of initial antibiotic treatment modification. The findings may precipitate reassessment of optimal management regimens for hospitalized CAP patients.

## Introduction

Community-acquired pneumonia (CAP) has an incidence rate of around 1 case per 1000 population per year in the EU [1] and is associated with considerable morbidity and mortality worldwide, with up to 68.8% of patients requiring hospitalization [1-4]. Previous studies show that patient outcomes are influenced by a number of factors, of which the decision to hospitalize and rapidity of initiation of antibiotic treatment are most important. The decision regarding site of care is critical [5,6] as low-risk patients may be vulnerable to worsened outcomes when treated in the hospital environment [6], and hospitalization for CAP is responsible for up to 80% of the total costs of this disease [7]. In bacterial pneumonia, rapid selection and initiation of appropriate antibiotic therapy is vital, shortening the illness course and significantly reducing the risk of complications or mortality [8]. Treatment decisions are complicated by the difficulty of obtaining a microbiological diagnosis, and empirical treatment, instead of pathogen-directed therapy, is standard [9]. Further, the ongoing development and shifting global patterns of antibiotic resistance may compromise effectiveness of previously useful antibiotics [10]. Finally, the choices of therapy available, both generic and branded, are numerous.

While the factors driving patient outcomes in CAP are increasingly understood, data on patient morbidity and mortality and associated resource use in Europe are scarce. Information is available mainly from individual countries rather than the whole continent and reports vary widely or are out of date [11]. Furthermore, comprehensive information on CAP management patterns in the real-life setting across Europe and their impact on patient outcomes is not available.

Therefore, we performed a retrospective observational study in ten EU countries (REACH; **Re**trospective Study to **A**ssess the **C**linical Management of Patients With Moderate-to-Severe Complicated Skin and Soft Tissue Infections (cSSTI) or CAP in the **H**ospital Setting) designed to create a better understanding of clinical management of these infections in response to current, real-world challenges. The study is a collaboration involving independent experts in CAP or cSSTI, a health economist, and clinical investigators across Europe, funded by AstraZeneca. The cSSTI data are reported separately elsewhere (Garau *et al.* submitted).

The CAP component reported here had two main objectives: to collect detailed background data on patients hospitalized with CAP in Europe, and to provide a summary of clinical practice decisions in these patients and their impact in terms of initial antibiotic treatment modification rates, associated length of hospital stay and mortality.

## Methods

### Overview

REACH ( http://NCT01293435) was a multinational, multicentre, observational, retrospective cohort study of patients hospitalized with CAP. Patients were enrolled from 128 sites in ten EU countries; Belgium, France, Germany, Greece, Italy, the Netherlands, Portugal, Spain, Turkey and the UK (see Additional file 1: Appendix 2 for full list of investigators). The study was performed according to Good Clinical Practice and the Declaration of Helsinki. All local ethics committees approved the study protocol. Local legislation relating to written informed consent for non-interventional studies was followed in each country; in Germany and Portugal, where this information is mandatory, written informed consent was collected.

### Patients

The population comprised patients with CAP requiring hospitalization identified between December 2010 and January 2011. All patients complying with relevant disease codes (Additional file 1: Appendix 1) in the World Health Organization International Classification of Diseases 10^th^ revision (ICD-10; 2007 version) were included [12]. The window for hospitalizations could be extended backward to March 2010 and forward to February 2011 until sufficient patients were identified. Patients to be included were selected from those identified by using an automatic randomization tool, in order to avoid selection bias.

#### Inclusion criteria

The study included adults (≥18 years of age) requiring treatment with intravenous (IV) antimicrobials. Radiographically confirmed pneumonia and acute illness (≤7 days’ duration) were required, with at least three of: new or increased cough; purulent sputum or change in sputum character; auscultatory findings consistent with pneumonia; dyspnoea, tachypnoea or hypoxaemia (O_2_ saturation <90% or pO_2_ <60 mmHg); fever (>38°C [oral]) or hypothermia (<35°C); white blood cell count >10,000 cells/mm^3^ or <4,500 cells/mm^3^; >15% band neutrophils irrespective of white blood cell count; requirement for initial hospitalization or treatment in an emergency room or urgent care setting.

#### Exclusion criteria

Patients already participating in a clinical trial or any other interventional study were not eligible. Patients with CAP deemed suitable for outpatient therapy with an oral antibiotic and patients transferred from another healthcare facility or readmitted with antibiotic use within 2 days were also excluded.

### Study variables

Data were collected via an electronic Case Report Form completed by the investigator. The information collected included site characteristics; patient demographics; medical history; disease characteristics, including severity score (Pneumonia Outcomes Research Team/Pneumonia Severity Index [PORT/PSI] [13]; CURB-65 [14]) and microbiological diagnosis; treatment setting; disease course and outcomes; antibacterial and other treatments before and during hospitalization and health resource consumption.

### Statistical methods and data interpretation

As the study is descriptive, no formal sample size calculations were performed. The aim was to recruit approximately 200–300 patients per disease per country to achieve a representative spread of patients.

The primary outcome measure was the initial antibiotic treatment modification rate. The initial antibiotic was the first IV antibiotic administered on admission to the hospital. ‘Initial antibiotic treatment modification’ was defined as a change in initial antibiotic treatment due to insufficient response, adverse reaction, interaction with other drugs, non-suitability of the initial antibiotic based on the results of microbiological tests or changes to or additions of new agents in a subsequent line, alone or in combination. Cases of streamlining (also known as de-escalation, defined as change to narrower-spectrum antibiotics upon patient improvement or confirmed microbiological diagnosis) were also recorded, but considered separately. Cases of patient death while on initial antibiotic treatment were also recorded.

Several antibiotic treatment modifications in the same patient were counted as a single case. Changes in dose or frequency of an existing antibiotic (considered as dose escalation or adaptation) and removal of an antibiotic from a combination and adaptation of the dose or frequency of the remaining antibiotic were not considered treatment modification.

For recording time to clinical stability, investigators were asked to report what criteria they had followed: Halm criteria [15], switch from IV to oral therapy or other criteria defined by the investigator.

## Results

### Patient population

**Figure 1:**
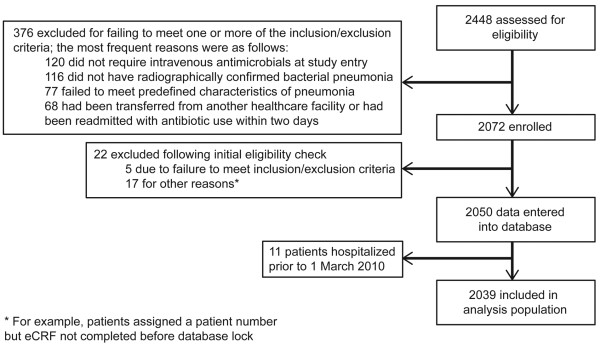
Patient flow.

The analysis population included 2,039 patients. Patient disposition is shown in Figure 1. The majority of patients were enrolled between November 2010 and February 2011.

Numbers of sites and numbers of patients per country are shown in Additional file 1: Table S1. Some countries did not achieve the target sample size of 200–300 patients; in Germany, the requirement for signed informed consent in a retrospective study precluded wider patient recruitment, leading to considerably fewer patients than other countries (n = 50), while in Portugal, changes in data protection laws delayed initiation, and in the UK, supply delays for some patient records necessitated their exclusion. Patient demographics and baseline characteristics are shown in Table 1.

**Table 1:** Patient demographics of analysis population

**Characteristic**	
Age, years, mean (SD) [median]	64.5 (18.5) [68.0]
≥65 years, n (%)	1,150 (56.4)
Female, n (%)	843 (41.3)
Ethnic origin, n (%)	
White	1,573 (77.1)
Non-white	51 (2.5)
Unknown/missing	51 (2.5)
Not applicable*	364 (17.9)
Residential/health status, n (%)	
Private house or apartment	1,720 (84.4)
Nursing home	145 (7.1)
Home care through healthcare agency	32 (1.6)
Previous admission to hospital with CAP (last 3 months)	99 (4.9)
Immunocompromised/immunosuppressed	72 (3.5)
Haemodialysis	6 (0.3)
Chemotherapy for active cancer	30 (1.5)
Other	43 (2.1)
Unknown	85 (4.2)
Smoking status, n (%)	
Non-smoker	704 (34.5)
Ex-smoker	553 (27.1)
Occasional smoker	42 (2.1)
Habitual smoker	463 (22.7)
Unknown	277 (13.6)

* All patients in this category were from France, where this question is not permitted in clinical studies. The discrepancy from the total number of patients for France (n = 366) arises because this information was actually recorded for two patients in France.

SD: standard deviation.

Medical history and disease characteristics are shown in Table 2. The mean age of patients differed considerably between patients with and without comorbidities (67.8 years and 52.6 years, respectively). Over half of the patients had received medications in the 3 months prior to admission.

**Table 2:** Medical history and disease characteristics

**Characteristic**	
Relevant medical conditions at hospitalization (index visit) (≥5% of analysis population), n (%)	
Any relevant condition	1,598 (78.4)
Respiratory disease	689 (33.8)
Diabetes	369 (18.1)
Congestive heart disease	336 (16.5)
Cancer/malignancy	237 (11.6)
Peripheral vascular disease	183 (9.0)
Renal disease	147 (7.2)
Other relevant conditions*	684 (33.5)
Medication history in the 3 months prior to hospitalization, n (%)	
Any prior medication	1,143 (56.1)
Antibiotics/antivirals	395 (19.4)
Anticoagulants	301 (14.8)
Immunosuppressants/immunomodulators	151 (7.4)
NSAIDs	137 (6.7)
Any other relevant therapies*	379 (18.6)
Unknown	146 (7.2)
Patient hospitalized for any reason in the 3 months prior to index visit, n (%)	204 (10.0)
Invasive surgical treatment in the 3 months prior to index visit, n (%)	33 (1.6)
Time since previous date of hospitalization, days, mean (SD) [median] (n = 182)	37.7 (24.0) [33.5]
Time since symptom start date to hospitalization (index visit), days, mean (SD) [median] (n = 1,905)	4.9 (9.1) [3.0]
Time since date of hospitalization (index visit) to first diagnosis date, days, mean (SD) [median] (n = 1,948)	0.5 (4.6) [0.0]

* As defined by the investigator.

NSAIDs: non-steroidal anti-inflammatory drugs; SD: standard deviation.

Characteristics of the index CAP infection are shown in Table 3. The majority of patients had no known recent contact with the healthcare system, having been resident in a private house or apartment prior to admission, while 12% of patients had been resident in settings commonly linked with healthcare-associated pneumonia (HCAP), such as in a nursing home. Antibiotic treatment of the index infection prior to hospitalization occurred in 23.5% of patients, the most common antibiotic classes used being penicillins or penicillin–β-lactamase inhibitor combinations (9.3%), fluoroquinolones (4.5%), cephalosporins (4.0%) and macrolides (3.5%).

**Table 3:** Characteristics of index CAP infection

**Characteristic**	
Type of CAP, n (%)	
CAP*	1,607 (78.8)
HCAP^†^	245 (12.0)
Immunocompromised/immunosuppressed	72 (3.5)
Other	43 (2.1)
Unknown	85 (4.2)
Radiographic findings suggestive of bacterial pneumonia, n (%)	
Infiltrate	1,168 (57.3)
Consolidation	947 (46.4)
Pleural effusion	319 (15.6)
Other	100 (4.9)
Unknown	16 (0.8)
Signs of acute illness at diagnosis, n (%)	
New or increased cough	1,575 (77.2)
Purulent sputum or change in sputum character	1,053 (51.6)
Auscultatory findings consistent with pneumonia	1,492 (73.2)
Dyspnoea, tachypnoea, or hypoxaemia	1,491 (73.1)
Fever or hypothermia	1,317 (64.6)
White blood cell count >10,000 cells/mm^3^ or <4,500 cells/mm^3^	1,352 (66.3)
Prognosis based on severity indices	
PORT/PSI	
Total, n (%)	354 (17.4)
Score, mean (SD)	3.5 (1.1)
I, n (%)	18 (0.9)
II, n (%)	53 (2.6)
III, n (%)	83 (4.1)
IV, n (%)	148 (7.3)
V, n (%)	52 (2.6)
CURB-65	
Number of patients (%)	527 (25.8)
Score, mean (SD)	2.2 (1.1)

* Residence in private house or apartment only.

^†^ Responses considered HCAP were: residence in a nursing home, home care through a healthcare agency, previous admission to hospital with CAP (last 3 months), haemodialysis or chemotherapy for active cancer, with the exception of immunocompromised/immunosuppressed.

CAP: community-acquired pneumonia; HCAP: healthcare-associated pneumonia; PORT/PSI: Pneumonia Outcomes Research Team/Pneumonia Severity Index; SD: standard deviation.

### Diagnostic information

Diagnostic information is shown in Additional file 1: Table S2. Although microbiological testing was conducted in all but one patient (Additional file 1: Table S2a), only 582 (28.5%) patients had a microbiological diagnosis available (Additional file 1: Table S2b). The most commonly isolated organism in the full analysis population was *Streptococcus pneumoniae* (39.2%). In patients with bacteraemia, this organism accounted for the majority of microbiological diagnoses (63.8%).

### Treatment setting and modalities

In total, 128 sites were included. A large number of university (teaching) hospitals were included (n = 54; 42.2%). Almost all sites were publicly funded (n = 124; 96.9%) and the majority were large (>500 beds: n = 91; 71.1%). Participating investigators were most commonly pneumologists (n = 63; 49.2%) or infectious diseases specialists (n = 35; 27.3%). Similar numbers of patients were treated in university and non-university hospitals (958 [47.0%] versus 1,081 [53.0%], respectively) and there were no major differences in patient population between hospital types. Overall use of the PORT/PSI and CURB-65 illness severity scoring systems was low and was more common in university hospitals than in non-university hospitals: PORT/PSI (30.3% versus 5.9%, respectively) and CURB-65 (39.0% versus 14.2%, respectively).

Antibiotics were most frequently administered on the first day of hospitalization (90.0% of patients; n = 1836), with 7.3% of patients (n = 149) receiving antibiotics on the second day. Antibiotics were administered empirically in 1,918 (94.1%) of patients. An analysis of antibiotic therapy administered is presented in Additional file 1: Table S3. Up to 48 different antibiotic agents (alone or in combination) were reported to have been used, the most frequent at initial line being amoxicillin-clavulanate (n = 409; 20.1%). The most common antibiotic families at initial line (whether used as monotherapy or in combinations) were penicillins or penicillin plus β-lactamase inhibitor combinations (54.7%), fluoroquinolones (29.0%), cephalosporins (29.5%) and carbapenems (1.8%). The mean overall treatment duration (calculated as start date of initial-line antibiotic to end date of last-line antibiotic) was 10.0 days (SD: 6.6; median: 9.0).

### Clinical outcomes

Clinical outcomes are shown in Table 4. The most common reasons for initial antibiotic treatment modification were insufficient response to treatment (12.0%) and adverse events (2.0%). In some patients, the reason recorded was ‘Other’ or ‘Unknown’, or no reason was reported. On case-by-case review by the investigators, these were found not to be related to clinical improvement, the availability of a microbiological diagnosis or streamlining. For patients in the ‘Other’, ‘Unknown’ and ‘No reason reported’ categories, time to antibiotic treatment modification was <4 days in 37.6% (n = 114) of patients, ≥4 days in 62.0% (n = 188) and unknown in 0.3% (n = 1). The median length of stay in hospital, including all hospitalizations for patients with recurrences, was 10.0 days (mean: 12.6 days; SD: 10.6). If recurrences were excluded, the median length of stay was 9.0 days (mean: 12.1 days; SD: 9.8).

**Table 4:** Clinical outcomes (full population)

**Outcome**	
Initial antibiotic treatment modification, n (%), for reasons:*	589 (28.9)
Insufficient response/treatment failure	244 (12.0)
Adverse events	41 (2.0)
Possible interaction with other treatment	1 (<0.1)
Other	149 (7.3)
Unknown	47 (2.3)
No reason reported	107 (5.2)
Death while on initial therapy	63 (3.1)
Streamlining (de-escalation) of therapy^†^, n (%)	105 (5.1)
Time to initial treatment modification, days, mean (SD) [median] (n = 760)	5.0 (3.8) [4.0]
Number of antibiotic therapy courses, n (%)	
1	1,086 (53.3)
2	644 (31.6)
3	190 (9.3)
>3	116 (5.7)
Time to clinical stability, days, mean (SD) [median] (n = 1,603)	5.6 (5.1) [4.0]
Based on Halm criteria (n = 588)	5.3 (5.4) [4.0]
Based on switch from IV to oral therapy (n = 738)	5.5 (4.1) [5.0]
Based on other criteria (n = 278)	6.4 (6.6) [5.0]
Length of stay, days, mean (SD) [median] (n = 1,978)^‡^	12.6 (10.6) [10.0]
Patients admitted to the ICU (n = 267)^§^	19.1 (17.1) [14.0]
Patients not admitted to the ICU (n = 1,691)^§^	10.9 (7.5) [9.0]
Discharged from hospital, n (%)	1,836 (90.0)
Reinfection/recurrence, n (%)^¶^	94 (4.6)
Home-based care after discharge, n (%)	73 (3.6)
Duration of home-based care, days, mean (SD) [median] (n = 38)	14.7 (15.0) [7.5]
Mortality rate, n (%)	147 (7.2)

* If multiple reasons are reported, the more clinically relevant reasons were selected first as the primary reason for change. The ordering of reasons from most relevant to least relevant are: Adverse event, Insufficient response/treatment failure, Possible interaction with other treatment, Other, Unknown.

^†^ De-escalation of treatment to narrower-spectrum antibiotics upon patient improvement or confirmed microbiological diagnosis.

^‡^ Includes duration of all hospitalizations for patients with recurrences.

^§^ Does not include duration of all hospitalizations for patients with recurrences.

^¶^ Refers to patients hospitalized again (due to CAP) after initial discharge.

IV: intravenous; SD: standard deviation.

**Figure 2:**
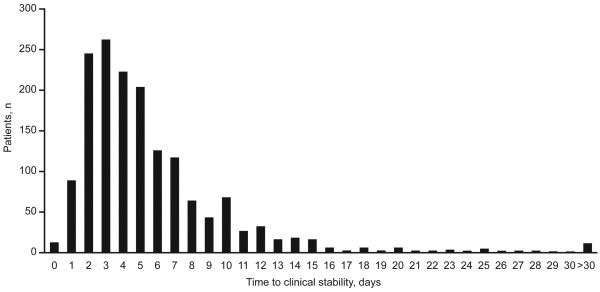
Distribution of patients according to time to clinical stability.

The distribution of time to clinical stability according to any of the criteria is shown in Figure 2. The majority of patients reached clinical stability on days 2–5. Approximately half of the patients with clinical stability data (n = 1,604) achieved clinical stability early in the course of treatment (day ≤4) (51.9%; n = 833), and 97.1% had achieved clinical stability on or before day 15. However, a small percentage (0.7%) of patients had not achieved clinical stability by day 30. Clinical failure (defined as acute haemodynamic deterioration or death, or any other criterion considered by the investigator to be indicative of clinical failure) occurred in 355 patients (17.4%). Of these, the failure was related to CAP in 239 patients (67.3%), unrelated in 85 patients (23.9%) and for unknown reasons in 31 patients (8.7%).

Initial antibiotic treatment modification rates by initial antibiotic agent for the most common antibiotic combinations and monotherapies are shown in Additional file 1: Table S3. Rates differed widely between antibiotics, with no obvious pattern. Clinical outcomes according to disease characteristics are shown in Table 5. Initial antibiotic treatment modification rates were greater in patients with HCAP (31.8%) and immunocompromised/immunosuppressed patients (40.3%) than in patients with CAP (28.4%). Mortality rates were also higher in these subgroups (HCAP: 16.3%; immunocompromised/immunosuppressed: 9.7%) compared with CAP (5.5%). Similarly, high initial antibiotic treatment modification rates were observed in patients with comorbidities versus those without. Worsened clinical outcomes were observed with increased severity of illness as measured by CURB-65 and PORT/PSI scores, albeit with small patient numbers. For patients with vaccine data recorded, neither influenza vaccination nor pneumococcal vaccination had a significant impact on outcomes. Mortality for the full population was 7.2%. Mortality varied across the different countries; Belgium, 12.0% (n = 23/191); France, 5.2% (n = 19/366); Germany 0% (n = 0/50); Greece 2.3% (n = 5/215); Italy, 1% (n = 3/300); the Netherlands, 10.8% (22/203); Portugal, 15.7% (n = 19/121); Spain, 6.8% (n = 19/279); Turkey, 8.5% (n = 17/200) and the UK 17.5% (n = 20/114).

**Table 5:** Clinical outcomes according to disease characteristics

**Outcome**	**Type of CAP**			**Recurrence**		**Comorbidities**		**Severity**							
								**PORT/PSI risk score**					**CURB-65 risk groups**		
	**CAP only (n = 1,607)**	**HCAP (n = 245)**	**Immuno-compromised/immuno-suppressed (n = 72)**	**With (n = 94)**	**Without (n = 1,548)**	**With (n = 1,598)**	**Without (n = 441)**	**I (n = 18)**	**II (n = 53)**	**III (n = 83)**	**IV (n = 148)**	**V (n = 52)**	**Mild (0–1) (n = 154)**	**Moderate (2) (n = 185)**	**Severe (3–5) (n = 176)**
Initial antibiotic treatment modification, n (%)	456 (28.4)	78 (31.8)	29 (40.3)	32 (34.0)	409 (26.4)	473 (29.6)	116 (26.3)	6 (33.3)	15 (28.3)	19 (22.9)	42 (28.4)	19 (36.5)	37 (24.0)	47 (25.4)	55 (31.3)
Streamlining, n (%)	72 (4.5)	21 (8.6)	9 (12.5)	8 (8.5)	82 (5.3)	84 (5.3)	21 (4.8)	0	4 (7.5)	6 (7.2)	6 (4.1)	4 (7.7)	4 (2.6)	7 (3.8)	6 (3.4)
Reinfection/recurrence, n (%)	70 (4.4)	16 (6.5)	5 (6.9)	94 (100)	0	88 (5.5)	6 (1.4)	0	2 (3.8)	4 (4.8)	9 (6.1)	5 (9.6)	2 (1.3)	11 (5.9)	12 (6.8)
Length of stay, days, median	9.0 (n = 1,558)	10.0 (n = 235)	12.0 (n = 71)	11.0 (n = 94)	9.0 (n = 1,548)	10.0 (n = 1,555)	8.0 (n = 423)	8.0 (n = 18)	8.0 (n = 53)	10.0 (n = 80)	10.0 (n = 148)	13.0 (n = 51)	8.0 (n = 153)	9.5 (n = 180)	10.0 (n = 175)
Time to clinical stability, days, median	4.0 (n = 1,284)	4.0 (n = 182)	5.0 (n = 55)	5.0 (n = 78)	4.0 (n = 1,295)	4.0 (n = 1,258)	4.0 (n = 345)	3.5 (n = 18)	3.0 (n = 43)	4.0 (n = 63)	5.0 (n = 139)	7.0 (n = 40)	4.0 (n = 137)	5.0 (n = 156)	5.0 (n = 142)
Mortality rate, n (%)	89 (5.5)	40 (16.3)	7 (9.7)	1 (1.1)	1 (0.1)	128 (8.0)	19 (4.3)	0	1 (1.9)	7 (8.4)	6 (4.1)	11 (21.2)	6 (3.9)	12 (6.5)	29 (16.5)

CAP: community-acquired pneumonia; HCAP: healthcare-associated pneumonia; PORT/PSI: Pneumonia Outcomes Research Team/Pneumonia Severity Index; SD: standard deviation.

### Clinical outcomes in patients attending recurrently with the same infection

In 94 patients (4.6%), the index CAP infection was a recurrence of a previously hospitalized CAP episode. The initial antibiotic treatment modification rate in these patients was 34.0% (n = 32). The median duration of hospital stay was 11.0 days (n = 94) and the median time to clinical stability was 5.0 days (n = 78) (Table 5).

## Discussion

CAP-associated morbidity and mortality are considerable and particularly common in patients hospitalized with CAP [16]. The REACH study gathered data on underlying characteristics and treatment patterns in patients hospitalized with CAP in a variety of clinical settings across 128 hospitals in ten EU countries. We found an unexpectedly high overall rate of initial antibiotic treatment modification (28.9%), with the majority of the patients treated empirically. Rates of initial antibiotic treatment modification and associated outcomes, such as overall mortality, were increased in patients with more complicated or more severe illness, such as those patients with a PORT/PSI score of V or ‘severe’ CURB-65 score, immunocompromised/immunosuppressed patients and patients with recurrent infections or comorbidities. Additionally, patients with HCAP had a much higher mortality rate of 16.3% compared with 5.5% in patients with CAP. Total mortality in REACH appears low at 7.2% but is consistent with data published previously for patients with PORT scores [17,18]. However, it may be that REACH patients without a measure of severity (PORT/PSI or CURB-65) actually had less severe disease globally.

Comparison with previous studies is complicated because different definitions of treatment modification and treatment failure are employed. Indeed, a 2009 review of treatment failure in patients with CAP across Europe found a wide variation (2.4–31%) in reported rates (including both early and late failure) [19]. In studies with treatment failure defined as per the definition of initial antibiotic treatment modification used in REACH, treatment failure rates align closely with the rate of initial antibiotic treatment modification observed [17,18].

A health economic analysis of our study showed that initial antibiotic treatment modification is associated with higher use of resources compared with no modification of initial antibiotic treatment (Ostermann *et al.* submitted). Therefore, it is critically important that management decisions for patients with CAP incorporate measures that reduce the likelihood of initial antibiotic treatment modification, including selection and rapid initiation of the most effective antibiotic agent, efficient diagnostic methods and early identification of patients with additional concerns. Initiatives aimed at improving empiric treatment strategies and microbiological stewardship may help in this respect.

The broad range of potential pathogens implicated in CAP and the difficulty in obtaining a precise microbiological diagnosis complicate treatment of CAP. In this study, initial treatment decisions were empiric in 94.1% of patients. A further complication is the wide range of treatment options: 48 different antibiotic regimens were reported in this study.

Although almost every patient underwent a microbiological test, the diagnosis rate was only 28.5%. The most common organism identified was *S. pneumoniae*, which follows previous studies [8,20-24]. Interestingly, high percentages of less common pathogens, such as *S. aureus* (7.2%) and *Pseudomonas aeruginosa* (7.0%), were observed. A Swedish study found that these pathogens were more common in patients with higher CRB-65 scores [20], and a European review of patients with CAP admitted to intensive care [25] observed *S. aureus* (7.0%) and Gram-negative enteric bacilli (8.6%) more frequently than in the whole population [8]. In patients with bacteraemia in REACH, the spectrum of pathogens was more homogeneous, being dominated by *S. pneumoniae* (n = 74; 63.8%). Compared with previous studies [26,27], a low proportion of resistant pathogens was reported in our study (two patients with penicillin-resistant *S. pneumoniae* [0.3%] and 12 with methicillin-resistant *S. aureus* [2.1%]).

Previous studies show that adherence to guidelines for antibiotic therapy for CAP [6,28] can reduce hospital length of stay [29,30], costs [30,31] and mortality [32]. The European Respiratory Society (ERS), in collaboration with The European Society for Clinical Microbiology and Infectious Disease (ESCMID), recommends penicillins, with or without β-lactamase inhibitors, or cephalosporins, administered in combination with newer macrolides for patients hospitalized with CAP [5]. Although there were a large number of different initial antibiotic regimens used in REACH, the majority of treatment decisions were consistent with these guidelines.

Interestingly, several of the most common antibiotic classes (penicillins plus macrolide, 32.3%; cephalosporin [excluding cefuroxime] plus fluoroquinolone, 31.3%) were associated with initial antibiotic treatment modification rates slightly higher than the average for all treatments. This may reflect inappropriate initial treatment choices for the infecting pathogen. A further possible explanation is higher use of certain antibiotics in patients with more severe illness, who may have been predisposed to initial antibiotic treatment modification. It should be noted that the initial antibiotic treatment modification rates reported for antibiotic classes may not generalize to individual agents within that class.

Our study had a number of limitations. The retrospective design may have resulted in inconsistent outcomes assessment between investigators due to differences in interpretation. However, the potential problem of incomplete information in some patient records was not common, generally occurring in ≤7% of patients (although the reason for initial antibiotic treatment modification was ‘Other’, ‘Unknown’ or was not reported in 14.8% of patients). The small patient numbers in some of the subgroups limit the possibility of making firm conclusions. Patient recruitment varied widely between countries owing to differences in ethical requirements. Also, the included countries were mainly western European, and so may not be fully representative of Europe as a whole.

In summary, this large, Europe-wide study provides the most current data to further describe patient characteristics and clinical management of patients hospitalized with CAP in this region. The findings reveal the enormous heterogeneity in clinical management patterns. Initial antibiotic treatment modification occurred in almost one-third of affected patients, and was more common in patients with comorbidities than in those without. Therefore, the authors believe that a reassessment of optimal management regimens should be undertaken and new therapies may be required to address this unmet need.

## Competing interests

FB has received research grants from Chiesi, GSK, Pfizer and Zambon, has received congress lecture fees from Abbott, Chiesi, GSK and Pfizer and has received consultancy fees from AstraZeneca, GSK and Pfizer. JG has received research grants, speaking invitations and conference invitations from Astellas, AstraZeneca, Bayer, GSK, Novartis, Pfizer and Vifor Pharma, and has recent or ongoing consultancies with Astellas, AstraZeneca, Bayer, Durata, GSK, Janssen Cilag, Novartis, Pfizer, Theravance and Vifor Pharma. HO is a member of an advisory board for AstraZeneca. JM and MA are employees of AstraZeneca. KMB has received consultancy fees from ACT Oncology, AstraZeneca, BioSoteria, Celgene Corporation, Cypress Pharmaceuticals, Integrium LLC, Outcomes Research (now owned by Quintiles), MedImmune, Multiple Myeloma Research Foundation, Sigma-Tau Pharmaceuticals and Worldwide Clinical Trials.

## Authors’ contributions

The chief investigators (JG, FB, HO) designed the trial, with input from the sponsor. The chief investigators, together with KM, initiated the analysis presented here, with the other investigators, JM and MA contributing to the analysis and interpretation. The decision to submit the report for publication was made by the lead contributors and chief investigators, who drafted and finalized the report with the help of a medical writer. The sponsor funded editorial assistance and reviewed the draft before submission. All authors have read and approved the final manuscript.

## Supplementary Material

Additional file 1: Table S1 Analysis population by country. **Table S2** Microbiological diagnosis. **Table S3** Antibiotic therapies. **Table S4** Patient demographics in patients with initial antibiotic treatment modification.** Table S5** Medical history and disease characteristics.** Table S6** Characteristics of index CAP infection. **Appendix 1** Pneumonias – ICD-10 coding. **Appendix 2** REACH study investigators. Click here for file
