# Supplementary material for: Current management of patients hospitalized with community-acquired pneumonia across Europe: outcomes from REACH
Source: Respir Res. 2013 Apr 15;14(1):44. doi: 10.1186/1465-9921-14-44 (PMC3644236; doi:10.1186/1465-9921-14-44)
Supplement: Additional file 1: Table S1 — Analysis population by country. Table S2 Microbiological diagnosis. Table S3 Antibiotic therapies. Table S4 Patient demographics in patients with initial antibiotic treatment modification. Table S5 Medical history and disease characteristics. Table S6 Characteristics of index CAP infection. Appendix 1 Pneumonias – ICD-10 coding. Appendix 2 REACH study investigators. [file 1465-9921-14-44-S1.pdf]

Additional Table 1. Analysis population by country

**Country**	**Number of sites**	**Patients included in analysis population, n (% total analysis population)**
Belgium	10	191 (9.4)
France	35	366 (17.9)
Germany	7	50 (2.5)
Greece	9	215 (10.5)
Italy	17	300 (14.7)
The Netherlands	6	203 (10.0)
Portugal	8	121 (5.9)
Spain	17	279 (13.7)
Turkey	11	200 (9.8)
United Kingdom	8	114 (5.6)
**Total**	**128**	**2,039 (100)**

Additional Table 2. Microbiological diagnosis

2a) Tests performed

**Test**	**Patients, n (%)**
Any microbiological test (cultures)	2,038 (>99.9)
Sputum examination	921 (45.2)
Blood culture	1121 (55.0)
Bronchoalveolar lavage/bronchial brush examination	177 (8.7)
Pleural fluid sample examination	91 (4.5)
Legionella antigen test in urine	785 (38.5)
Pneumococcal antigen test in urine	713 (35.0)
Other	378 (18.5)
Unknown	308 (15.1)
PCR determination of H1N1 influenza performed, n (%)	351* (17.2)
Positive	76 (3.7)
Negative	276 (13.5)

* Two patients reported "No" to whether determination was performed, but had "Negative" recorded for the result; one patient reported "Yes" to whether determination was performed but the result was not given. Therefore, there is one more result than the number of patients for which the test was reported as performed.

2b) Diagnosis

**Microbiological diagnosis, n (percentage of patients with a microbiological diagnosis, %)**	**Total population (n=582; 28.5%)**	**Patients with bacteraemia (n=116; 5.7%)**
Gram-positive cocci	286 (49.1)	83 (71.6)
Streptococcus pneumoniae	228 (39.2)	74 (63.8)
Penicillin-resistant *Streptococcus pneumoniae**	2 (0.3)	1 (0.9)
Staphylococcus aureus†	42 (7.2)	9 (7.8)
Methicillin-resistant *Staphylococcus aureus**	12 (2.1)	2 (1.7)
Methicillin-sensitive *Staphylococcus aureus*	9 (1.5)	0 (0.0)
Legionella spp.	20 (3.4)	0 (0.0)
Mycoplasma pneumoniae	18 (3.1)	0 (0.0)
Chlamydophila pneumoniae	13 (2.2)	0 (0.0)
Chlamydia spp.	0 (0.0)	0 (0.0)
Haemophilus influenzae	33 (5.7)	5 (4.3)
Haemophilus parainfluenzae	6 (1.0)	1 (0.9)
Moraxella catarrhalis	9 (1.5)	0 (0.0)
Pseudomonas aeruginosa	41 (7.0)	2 (1.7)
Enterobacteriaceae	91 (15.6)	13 (11.4)
Escherichia coli	36 (6.2)	10 (8.6)
Klebsiella spp.	20 (3.4)	1 (0.9)
Other enterobacteria‡	35 (6.0)	2 (1.7)
Aspiration pneumonia	17 (2.9)	2 (1.7)
Other microorganisms	133 (22.9)	17 (14.7)

* Minimum inhibitory concentration data not available retrospectively.

† Not all *S. aureus* infections had susceptibility data recorded, so the total *S. aureus* figure exceeds the total of the resistant and sensitive figures.

‡ Includes *Proteus mirabilis*, *Morganella morganii*, *Serratia marcescens* and *Hafnia alvei*.

Additional Table 3. Antibiotic therapies

**Antibiotic**	**Initial line, n (%)**	**Subsequent lines, n (%)**	**Rate of initial antibiotic treatment modification, n (%)**
Combinations			
Penicillin or penicillin–β-lactamase inhibitor + macrolide	288 (14.1)	20 (1.0)	93 (32.3)
Cephalosporin (except cefuroxime) + macrolide	211 (10.3)	10 (0.5)	55 (26.1)
Cephalosporin (except cefuroxime) + fluoroquinolone	140 (6.9)	10 (0.5)	44 (31.4)
Penicillin or penicillin–β-lactamase + fluoroquinolone	115 (5.6)	25 (1.2)	36 (31.3)
Monotherapies			
Amoxicillin–clavulanate	409 (20.1)	274 (13.4)	100 (24.4)
Levofloxacin	156 (7.7)	98 (4.8)	34 (21.8)
Moxifloxacin	116 (5.7)	57 (2.8)	20 (17.2)
Ceftriaxone	109 (5.3)	42 (2.1)	42 (38.5)
Piperacillin–tazobactam	66 (3.2)	75 (3.7)	24 (36.4)
Amoxicillin	59 (2.9)	64 (3.1)	9 (15.3)
Clarithromycin	19 (0.9)	41 (2.0)	6 (31.6)
Azithromycin	12 (0.6)	43 (2.1)	4 (33.3)
Cefuroxime	26 (1.3)	28 (1.4)	11 (42.3)
Ampicillin–sulbactam	36 (1.8)	9 (0.4)	15 (41.7)
Ciprofloxacin	9 (0.4)	37 (1.8)	5 (55.6)
Meropenem	6 (0.3)	38 (1.9)	2 (33.3)
Vancomycin	1 (<0.1)	23 (1.1)	0

Characteristics of patients with initial treatment modification

We conducted an analysis of patient characteristics in the subpopulation of patients with initial antibiotic treatment modification (589) (Supplementary Tables 4–6). No clear differences between patients with initial antibiotic treatment modification and the full population were observed in terms of demographics, medical history and characteristics of index CAP infection. Comorbidities were slightly more common in patients with initial antibiotic treatment modification than in the full population (80.3% versus 78.4%, respectively), and HCAP was present in more patients (13.2% versus 12.0%, respectively).

Additional Table 4. Patient demographics in patients with initial antibiotic treatment modification

**Characteristic**	**Full population (N=2,039)**	**Patients with initial antibiotic treatment modification (n=589)**
Age, years, mean (SD) [median]	64.5 (18.5) [68.0]	64.2 (18.7) [68.0]
≥65 years, n (%)	1,150 (56.4)	330 (56.0)
Female, n (%)	843 (41.3)	226 (38.4)
Ethnic origin, n (%)		
White	1,573 (77.1)	486 (82.5)
Non-white	51 (2.5)	22 (3.7)
Unknown/missing	51 (2.5)	24 (4.1)
Not applicable*	364 (17.9)	57 (9.7)
Residential/health status, n (%)		
Private house or apartment	1,720 (84.4)	495 (84.0)
Nursing home	145 (7.1)	47 (8.0)
Home care through healthcare agency	32 (1.6)	11 (1.9)
Previous admission to hospital with CAP (last 3 months)	99 (4.9)	33 (5.6)
Immunocompromised/immunosuppressed	72 (3.5)	29 (4.9)
Haemodialysis	6 (0.3)	2 (0.3)
Chemotherapy for active cancer	30 (1.5)	12 (2.0)
Other	43 (2.1)	12 (2.0)
Unknown	85 (4.2)	20 (3.4)
Smoking status, n (%)		
Non-smoker	704 (34.5)	181 (30.7)
Ex-smoker	553 (27.1)	164 (27.8)
Occasional smoker	42 (2.1)	11 (1.9)
Habitual smoker	463 (22.7)	132 (22.4)
Unknown	277 (13.6)	101 (17.1)

CAP: community-acquired pneumonia; SD: standard deviation.
* All patients in this category were from France, where this question is not permitted in clinical studies. The discrepancy from the total number of patients for France (n=366) arises because this information was actually recorded for two patients in France.

Additional Table 5. Medical history and disease characteristics

**Characteristic**	**Full population (N=2,039)**	**Patients with initial antibiotic treatment modification (n=589)**
Relevant medical conditions at hospitalization (index visit) (≥5% of analysis population), n (%)		
Any relevant condition	1,598 (78.4)	473 (80.3)
Respiratory disease	689 (33.8)	192 (32.6)
Diabetes	369 (18.1)	103 (17.5)
Congestive heart disease	336 (16.5)	102 (17.3)
Cancer/malignancy	237 (11.6)	80 (13.6)
Peripheral vascular disease	183 (9.0)	51 (8.7)
Renal disease	147 (7.2)	46 (7.8)
Other relevant conditions*	684 (33.5)	210 (35.7)
Medication history in the 3 months prior to hospitalization, n (%)		
Any prior medication	1,143 (56.1)	351 (59.6)
Antibiotics/antivirals	395 (19.4)	138 (23.4)
Anticoagulants	301 (14.8)	82 (13.9)
Immunosuppressants/immunomodulators	151 (7.4)	49 (8.3)
NSAIDs	137 (6.7)	41 (7.0)
Any other relevant therapies*	379 (18.6)	123 (20.9)
Unknown	146 (7.2)	40 (6.8)

NSAIDs: non-steroidal anti-inflammatory drugs.
* As defined by the investigator.

Additional Table 6. Characteristics of index CAP infection

**Characteristic**	**Full population (N=2,039)**	**Patients with initial antibiotic treatment modification (n=589)**
Type of CAP, n (%)		
CAP*	1,607 (78.8)	456 (77.4)
HCAP†	245 (12.0)	78 (13.2)
Immunocompromised/immunosuppressed	72 (3.5)	29 (4.9)
Other	43 (2.1)	12 (2.0)
Unknown	85 (4.2)	20 (3.4)
Radiographic findings suggestive of bacterial pneumonia, n (%)		
Infiltrate	1,168 (57.3)	334 (56.7)
Consolidation	947 (46.4)	277 (47.0)
Pleural effusion	319 (15.6)	111 (18.8)
Other	100 (4.9)	32 (5.4)
Unknown	16 (0.8)	2 (0.3)
Signs of acute illness at diagnosis, n (%)		
New or increased cough	1,575 (77.2)	436 (74.0)
Purulent sputum or change in sputum character	1,053 (51.6)	271 (46.0)
Auscultatory findings consistent with pneumonia	1,492 (73.2)	423 (71.8)
Dyspnoea, tachypnoea or hypoxaemia	1,491 (73.1)	447 (75.9)
Fever or hypothermia	1,317 (64.6)	381 (64.7)
White blood cell count >10,000 cells/mm^3 or <4,500 cells/mm^3	1,352 (66.3)	391 (66.4)
Prognosis based on severity indices		
PORT/PSI		
Number of patients (%)	354 (17.4)	101 (17.1)
Score, mean (SD) [median; range]	3.5 (1.1) [4.0; 1–5]	3.5 (1.1) [4.0; 1–5]
CURB-65		
Number of patients (%)	527 (25.8)	147 (25.0)
Score, mean (SD) [median; range]	2.2 (1.1) [2.0; 1–5]	2.3 (1.1) [2.0; 1–5]

CAP: community-acquired pneumonia; HCAP: healthcare-associated pneumonia; PORT/PSI: Pneumonia Outcomes Research Team/Pneumonia Severity Index; SD: standard deviation.
* Residence in private house or apartment only.
† Responses considered HCAP were all other residential statuses with the exception of immunocompromised/immunosuppressed.

APPENDIX 1. Pneumonias – ICD-10 coding

**A48.1**	Legionnaires' disease
**J13**	Pneumonia due to *Streptococcus pneumoniae*
**J14**	Pneumonia due to *Haemophilus influenzae*
**J15.0**	Pneumonia due to *Klebsiella pneumoniae*
**J15.1**	Pneumonia due to *Pseudomonas* sp.
**J15.20**	Pneumonia due to *Staphylococcus* sp., unspecified
**J15.21**	Pneumonia due to *Staphylococcus aureus*
**J15.3**	Pneumonia due to *Streptococcus* sp., group B
**J15.4**	Pneumonia due to other streptococci
**J15.5**	Pneumonia due to *Escherichia coli*
**J15.6**	Pneumonia due to other aerobic Gram-negative bacteria
**J15.7**	Pneumonia due to *Mycoplasma pneumoniae*
**J15.8**	Pneumonia due to other specified bacteria
**J15.9**	Unspecified bacterial pneumonia
**J16.0**	Chlamydial pneumonia
**J16.8**	Pneumonia due to other specified infectious organisms
**J18.0**	Bronchopneumonia, unspecified organism
**Z16**	Infection with drug-resistant microorganisms

APPENDIX 2 - REACH study investigators

Belgium; Frédérique Jacobs, Clinique Universitaire Bruxelles Hôpital Erasme, Bruxelles; Rudi Peché, CHU Vésale, Montigny-le-Tilleul; Mickael Aoun, Institut Jules Bordet, Bruxelles; Bénédicte Delaere, Clinique Universitaire UCL Mont Godinne, Godinne; Camelia Rossi, CHU Ambroise Paré, Mons; Soraya Cherifi, CHU Brugmann, Bruxelles; Ignace Demeyer, OVL Aalst, Aalst; Ingeborg Gyssens, Jessa Ziekenhuis, Hasselt; Bernard Vandercam, UCL Saint-Luc, Bruxelles; Michel Moutschen, CHU Liège, Liège; Frédéric Forêt, CH de Dinant, Dinant; Philippe Jorens, UZ Antwerpen, Antwerpen; Chris Vercammen, AZ Imelda , Bonheiden; Remy Demeester, CHU de Charleroi, Charleroi. **France;** Karim Lachgar, Hôpital Simone Veil Eaubonne; Daniel Epain, CH de Lagny Sur Marne; Richard Naldjinan, Hopital Andrée Rosemon, Cayenne; Fréderic Lucht Hôpital Nord, Saint Priest en Jarez; Youssef El Samad, Hôpital Nord, Amiens; Houcine Bentayeb, Youcef Douadi and Charles Dayen, CH de Saint Quentin; Juliana Darasteanu, CH Louis Pasteur, Le Coudray; Veronique Rémy, CH Jean Rougier, Cahors; Caroline Dehecq, CH de Dunkerque; Emmanuel Forestier, CH de Chambery; Stephane Chadapaud, CH Marie Josée Treffot, Hyères; Jean Paul Stahl and Maxime Maignan, Hôpital Albert Michallon, La Tronche; Alain Puidupin, Hia Alphonse Laveran, Marseille; Abderrahim Labzour, CH Sud Ardennes de Rethel; Pascal Bilbault, Hôpital Hautepierre, Strasbourg; Gabriel Stoica Ion, CH Pierre Delpech, Decazeville; Didier Debieuvre, CHI de La Haute Saône de Vesoul; Cristina Hotea, CH de Tulle; Guillaume Leveiller, Hôpital Yves Le Foll, Saint Brieux; Laurent Portel, CH Samuel Pozzi, Bergerac; Gerard Lamarque, CH Laennec, Creil; Khaldoun Naaman, CH Francois Mitterrand, Pau; Jonny Nehme, CH De Vendôme; Immad Abdoush, Centre De Soins Polyvalent, Agde; Fréderic Girard, CH Des Escartons, Briançon; Nidal Mourad Agha, CH de Denain; Cecilia Nocent Ejnaini, Chic Cote Basque Bayonne; Benjamin Planquette, CH Andre Mignot, Le Chesnay; Fredj Mouez Harzallah, CHI Du Pays Des Hautes Falaises Fécamp; Sébastien Kasseyet, CH de Salon de Provence; Bruno Lemmens, CH D Amboise; Jean Damien Ricard, Hôpital Louis Mourier, Colombes; Anne Prudhomme, CH de Bigorre Site La Gespe, Tarbes; Mahdi Ould, CHI Elbeuf; Fedjer Salim Taazibt, CH de Montdidier; Malcolm Lemyze, CH Du Docteur Schaffner, Lens, Pau; Arnaud Scherpereel, Hôpital Calmette, Lille; Bruno Abraham, CH de Brive La Gaillarde; Wassim Bechlawi, CH de St Hilaire Du Harcouet; Pierre Sadoul, CHI D Alencon; Lucien Bernabeu, CH de Chauny; Abdellatif Hajjaji, CH Du Mans; Marie Agnes Vincent, CH Saint Charles, Toul; Etienne Devin, CHI Eure Seine D'Evreux; Stéphanie Dehette, CH de Compiègne; Nadine Paillot, Hôpital N D Bon Secours, Metz; Youssef El Far, CH de Saintonge; Jean François Vigneau, Hôtel Dieu, Paris. **Germany;** Hans-Christian Buschmann, Brüderkrankenhaus St. Josef Paderborn; Christian Schumann, Universitätsklinik Ulm; Tom Schaberg, Diakoniekrankenhaus Rotenburg gGmbH; Norbert Suttorp, Charité Berlin; Jürgen Behr, Berufsgenossenschaftliche Universitätsklinik Bochum; Stefan Krüger, Universitätsklinik Aachen; Wulf Pankow, Vivantes Klinikum Berlin Neukölln; Bernhard Schaaf, Klinikum Dortmund gGmbH; Peter Kujath, Universitätsklinikum Schleswig-Holstein, Lübeck; Frank Siemers, Universitätsklinikum Schleswig-Holstein, Lübeck; Matthias Geenen, Lubinus-Clinicum, Kiel. **Greece;** Niki Georgatou, Mina Gaga, Aggeliki Rapti and Ioannis Ntanos, Sotiria Hospital, Athens; Nikolaos Siafakas, University Hospital of Heraklion, Crete; Marianna Kakoura and Spiros Miyakis, Papageorgiou Hospital, Thessaloniki; Spyridon Papiris and Georgios Petrikkos, Attikon University Hospital, Athens; Konstantinos Gourgoulianis, University Hospital, Larissa; Georgios Chrysos, Tzaneio General Hospital, Piraeus; Panagiotis Gargalianos, Gennimatas General Hospital, Athens; Moyssis Lelekis, Amalia Fleming Hospital, Athens; Athanasios Skoutelis, Evangelismos Hospital, Athens; Efstratios Maltezos, University Hospital, Alexandroupolis; Thomas Gerasimidis and Athanasios Sakantamis, Hippokrateio Hospital, Thessaloniki. **Italy;** Luigi Fenoglio, Ospedale Santa Croce, Cuneo; Bruno Cacopardo, ARNAS Garibaldi Nuovo, Catania; Francesca Montagnani, Ospedali Civili Senese, Siena; Nicola Petrosillo, IRCCS Lazzaro Spallanzani, Roma; Arcangelo Iannuzzi, A. O. Cardarelli, Napoli; Domenico Caruso, A. O. Cardarelli, Napoli; Giampiero Carosi, Spedali Civili, Brescia; Michele Corritore, IRCCS Casa Sollievo della Sofferenza, San Giovanni Rotondo; Lucio Cosco, A. O. Pugliese Ciacco, Catanzaro; Alessandro Bartoloni, A. O. Careggi, Firenze; Marco Anselmo, Ospedale San Paolo, Savona; Alberto Artom, Ospedale Santa Corona, Pietra Ligure; Giuliano Rizzardini, Ospedale Luigi Sacco, Milano; Enzo Petrelli, A. O. Pesaro; Giovanni Pittoni, Azienda Ospedaliera di Padova; Francesco Blasi, Ospedale Maggiore Policlinico Cà Granda, Milano; Francesco Menichetti, Ospedale Cisanello, Pisa; Marco Tinelli, Ospedale Maggiore Civile, Lodi; Giuseppe Foti, Presisio Ospedaliero, Reggio Calabria; Claudio Mastroianni, Ospedale S.M. Goretti, Latina; Michele Ciccarelli, IRCCS Istituto Clinico Humanitas, Rozzano Milano; Lorenzo Minoli, Policlinico San Matteo, Pavia; Antonello Malfitano, Policlinico San Matteo, Pavia; Giovanni Riccio, Ospedale Santa Corona, Pietra Ligure; Pietro Caramello, Ospedale Amedeo di Savoia, Torino; Giovanni Di Perri, Ospedale Amedeo di Savoia, Torino; Patrizia Mangiarotti, Policlinico San Matteo, Pavia; Giovanni Cassola, Ospedale Civile

Galliera, Genova; Roberto Esposito, A.O.U. di Modena; Eligio Pizzigallo, Ospedale SS. Annunziata, Chieti; Paolo Grossi, Ospedale Circolo Macchi, Varese; Vincenzo Vullo, Università la Sapienza, Roma; Mario Cottone, A.O. Villa Sofia Cervello, Palermo; Gennaro D'amico; A.O. Villa Sofia Cervello, Palermo; Piergiorgio Rabitti, A. O. Cardarelli, Napoli; Pierluigi Viale, Ospedale Sant'Orsola Malpighi, Bologna; Guido Greca, Nuovo Ospedale Umberto I, Enna; Sebastiano Costantino, Policlinico Campus Biomedico, Roma; Ercole Concia, Ospedale Borgo Roma; Verona. **Netherlands;** Wim G. Boersma, Medisch Centrum Alkmaar, Alkmaar; Ger T. Rijkers, St. Antonius Ziekenhuis, Nieuwegein; Jan Jelrik Oosterheert, UMC Utrecht, Utrecht; Remco S. Djamin, Amphia Ziekenhuis, Breda; Steven J.M. Gans, St. Jansdal Ziekenhuis, Harderwijk; Tjip S. Van der Werf, UMCG, Groningen; Erik Honig, LangeLand Huidkliniek, Zoetermeer; Remco Van Doorn, Leids Universitair Medisch Centrum, Leiden; Wim J.A. de Kort, Dermatologiepraktijk BV, Breda; Luc J.A. Strobbe, Canisius Wilhelmina Ziekenhuis, Nijmegen. **Portugal;** Mariano Velez, Hospital Rainha Santa Isabel-Centro Hospitalar Médio Tejo, Torres Novas; Nuno Bragança, Hospital Prof. Dr. Fernando da Fonseca, Amadora; Margarida Mota, Centro Hospitalar Vila Nova de Gaia; Pedro Póvoa, Hospital São Francisco Xavier –Centro Hospitalar Lisboa Ocidental, Lisboa; Isabel Almeida, Centro Hospitalar Tâmega e Sousa, Penafiel; Manuela VanZeller Monteiro, Centro Hospitalar Vila Nova de Gaia; João Estevens, Centro Hospitalar do Barlavento Algarvio, Portimão; Filipe Froes, Hospital Pulido Valente-Centro Hospitalar Lisboa Norte, Lisboa; Alberto Midões, Hospital de Viana- Unidade Local de Saúde do Alto Minho, Viana do Castelo. **Spain;** César Martínez Vázquez, Complejo Hospitalario Xeral-Cies de Vigo; José María Aguado, Hospital Universitario 12 de Octubre, Madrid; Javier Cobo, Hospital Universitario Ramón y Cajal II, Madrid; Emilio Bouza, Hospital General Universitario Gregorio Marañón, Madrid; José Ramón Paño Pardo, Hospital Universitario La Paz, Madrid; Juan Pablo Horcajada, Hospital del Mar, Barcelona; Javier Garau, Hospital Universitari Mútua de Terrassa; Eva Polverino, Hospital Clínic de Barcelona; Miguel Sabria Leal, Hospital Universitari Germans Trias i Pujol, Badalona; Jordi Carratala, Hospital Universitari de Bellvitge, Barcelona; Irene Gracia Ahufinger, Hospital Universitario Reina Sofía, Córdoba; Jesús Rodríguez Baños, Hospital Universitario Virgen Macarena II, Sevilla; Jerónimo Pachón, Hospital Universitario Virgen del Rocío, Sevilla; Juan Diego Ruiz Mesa, Hospital Regional Universitario Carlos Haya, Málaga; Efrén Sánchez Vidal, Complexo Hospitalario Universitario A Coruña; Melchor Riera, Hospital Universitari Son Espases, Mallorca; Isidro Rodríguez, CHU de Santiago, Santiago de Compostela; Rosa Oltra, Hospital Clínico Universitario de Valencia; Esperanza Merino, Hospital General Universitario de Alicante II; Alfonso Moreno, Hospital Universitario Central de Asturias, Oviedo; Manuel Gutiérrez Cuadra, Hospital Universitario Marqués de Valdecilla, Santander. **Turkey;** Abdullah Sayiner and Fezal Özdemir, Ege University Medical Faculty; Serhat Unal and Lutfu Coplu, Hacettepe University Medical Faculty; Numan Ekim and Dilek Arman, Gazi University Medical Faculty; Oguz Kilinc, Dokuz Eylül University Medical Faculty; Akin Kaya, Ankara University Medical Faculty; Ali Kadri Cirak, Suat Seren Training and Research Hospital; Adnan Yilmaz, Sureyyapasa Training and Research Hospital; Esra Kunt Uzaslan, Uludag University Medical Faculty; Hakan Gunen, Umraniye Training and Research Hospital; Aykut Cilli and Rabin Saba, Akdeniz University Medical Faculty; Zeynep Gunes Ozinal, Izzet Baysal University Medical Faculty; Fehmi Tabak, Istanbul Cerrahpasa Medical Faculty; Ilhan Ozgunes, Osmangazi University Medical Faculty; Hakan Leblebicioglu, Ondokuz Mayýs University Medical Faculty; Gonul Cicek Senturk, Diskapi Training and Research Hospital; Arif Atahan Cagatay, Istanbul University Medical Faculty; Rahmet Guner, Atatürk Training and Research Hospital; Hurrem Bodur, Ankara Numune TRH. **United Kingdom;** Anthony De Soyza, Freeman Hospital, Newcastle-upon-Tyne; Martin Wiselka, Leicester Royal Infirmary, Leicester; Adam Hill, Edinburgh Royal Infirmary, Edinburgh; Achyut Guleri, Blackpool Victoria Hospital, Blackpool; David Chadwick, James Cook University Hospital, Middlesbrough; Stephen Gordon, University Hospital Aintree/Royal Liverpool and Broadgreen University Hospital, Liverpool; Matthew Dryden, Royal Hampshire County Hospital, Winchester; Gavin Barlow, Castle Hill Hospital, Cottingham.
